# Changes of Physicochemical Properties and Immunomodulatory Activity of Polysaccharides During Processing of *Polygonum multiflorum* Thunb

**DOI:** 10.3389/fphar.2022.934710

**Published:** 2022-06-16

**Authors:** Donglin Gu, Ying Wang, Hongyu Jin, Shuai Kang, Yue Liu, Ke Zan, Jing Fan, Feng Wei, Shuangcheng Ma

**Affiliations:** ^1^ Institute for Control of Chinese Traditional Medicine and Ethnic Medicine, National Institutes for Food and Drug Control, Beijing, China; ^2^ School of Traditional Chinese Pharmacy, China Pharmaceutical University, Nanjing, China; ^3^ School of Traditional Chinese Medicine, Beijing University of Chinese Medicine, Beijing, China

**Keywords:** *Polygonum multiflorum* Thunb, polysaccharides, processing, fingerprint, immunomodulatory activity

## Abstract

The roots of *Polygonum multiflorum* Thunb (PM) have a long history of usage in traditional Chinese medicine and are still widely utilized today. PM in raw or processed form has different biological activities and is commonly used to treat different diseases. Polysaccharides are the main component of PM, and it is unclear whether their physicochemical properties and activities change after processing. In this study, the polysaccharides from thirty-one raw PM (RPMPs) and nine processed PM (PPMPs) were extracted, and the physicochemical properties and immunomodulatory activity *in vitro* of polysaccharide samples were evaluated. Results showed that RPMPs and PPMPs had significant differences in physicochemical properties. RPMPs and PPMPs were both composed of mannose, rhamnose, glucuronic acid, galacturonic acid, glucose, galactose, and arabinose. However, RPMPs and PPMPs had significant differences in their yields, molecular weight (*M*w), and the molar ratio of Glc/GalA (*p* < 0.05), which can be used to distinguish raw and processed PM. The fingerprint of monosaccharide composition was analyzed by chemometrics, and it was further demonstrated that Glc and GalA could be used as differential markers. The immunomodulatory activity assays indicated that RPMPs and PPMPs could significantly enhance phagocytosis and mRNA expression of cytokines in RAW 264.7 cells. In addition, the immunomodulatory activity of PPMPs with lower *M*w was significantly better than that of RPMPs. This study furthers the understanding of the polysaccharides from raw and processed PM and provides a reference for improving the quality standard of PM.

## 1 Introduction

The roots of *Polygonum multiflorum* Thunb (PM) have a long history of use in traditional Chinese medicine (TCM) and are commonly used in raw or processed form to treat different diseases (Chen et al., 2021). Studies have shown that PM mainly contains anthraquinones, stilbene glycosides, polysaccharides, phospholipids, and so on (Liu et al., 2018). According to TCM theory, raw PM has detoxifying, anti-swelling, anti-malarial, and laxative properties, while processed PM is effective in tonifying the liver and kidney, strengthening tendons and bones, and darkening hair (Yang et al., 2019). Raw and processed PM are widely used as medicines and health products in China, Japan, and Southeast Asia. In China, the processing methods of PM have been practiced since the Tang dynasty. Currently, there are three processing methods for PM in the 2020 edition of “Chinese pharmacopeia”, i.e., stew with black bean juice, steam with black bean juice, and steam with water.

It is widely accepted that there are many differences in chemical composition and efficacy between raw and processed PM (Liang et al., 2010). Since the 1990s, the hepatotoxicity and clinical safety of PM have especially come under scrutiny as reports of adverse liver reactions caused by PM and PM-containing preparations have increased (Park et al., 2001; Mazzanti et al., 2004; Panis et al., 2005; Shaw, 2010; Dong et al., 2015). It has been shown that the hepatotoxicity of PM can be reduced after processing (Yu et al., 2011; Lin et al., 2015). According to the TCM theory, proper pharmaceutical processing methods may reduce toxicity, increase effectiveness, and change the pharmacological effects (Li et al., 2017). Some previous studies have suggested that the changes in stilbene glycosides and anthraquinones during the processing of PM may be related to liver injury.

Polysaccharide is an important component of PM, which has immunomodulatory, anti-tumor, antioxidant, and other activities (Chen et al., 2012; Zhu et al., 2017; Qing Zhang et al., 2018; Luo et al.). In recent years, the structure and activity of polysaccharides from PM have been studied in some reports. For example, the alkali-extractable polysaccharide from raw PM activated splenocytes and peritoneal macrophages, causing significant immunomodulation activity (Qing Zhang et al., 2018). Luo et al. (2011) revealed that polysaccharides from raw PM possessed excellent antioxidant capacity against free radicals, lipid oxidation, and protein glycation. Xu et al. (2014, 2) purified the polysaccharide from raw PM, finding it was composed of rhamnose, arabinose, xylose, and glucose. Qing Zhang et al. (2018) obtained two purified polysaccharides from raw PM and found that acidic polysaccharide WPMP-2 had better immunomodulatory activity than neutral polysaccharide WPMP-1. In addition, they also pointed out that WPMP-2 had a more complex branching structure than WPMP-1 and speculated on the partial structure of WPMP-1 and WPMP-2. However, most of these studies focused on structural characterization and activity determination. Currently, there are no reports on whether polysaccharides change during processing and how this affects the activity, which may advance the study of its liver injury mechanism.

In this study, we collected samples of raw and processed PM from different regions and markets, after which we determined the physicochemical properties (neutral sugar content, uronic acid content, molecular weight, and monosaccharide composition) and immunomodulatory activity *in vitro*. Furthermore, the chemometrics methods were utilized to analyze the data from fingerprints and to effectively assess the differences in polysaccharides from raw and processed PM. These findings could serve as a benchmark for enhancing the quality control of PM and promote the research on the immunomodulatory mechanism of polysaccharides.

## 2 Materials and Methods

### 2.1 Materials and Reagents

Forty batches of raw PM (1–31) and processed PM (32–40) were collected from different locations and markets in China (Table 1). Associate Professor Shuai Kang (National Institute for Food and Drug Control, China) authenticated the samples of PM. Sample 31 was a raw product collected from Henan Province, while sample 39 was a processed product of sample 31 according to the 2020 edition of “Chinese pharmacopeia”. Sample 39 was processed as follows: the raw PM was mixed with black bean juice and steamed until it was brown inside and outside, then sliced and dried.

**TABLE 1:** Essential information of the 40 polysaccharide samples.

Codes	Origins	Yields (%)	Neutral sugar content (%)	Uronic acid content (%)	Protein content (%)	*M*w (kDa)	*M*w/*Mn*	Peak 2/Peak 1
1	Wenshan, Yunnan	3.46	52.68	6.64	19.67	1,126.10	3.12	3.63
2	Qujin, Yunnan	2.86	51.24	8.96	20.32	1,546.30	3.28	3.88
3	Qujin, Yunnan	2.05	40.39	10.26	13.20	1,011.70	3.49	3.55
4	Qujin, Yunnan	3.98	43.98	7.37	21.92	1,464.70	3.25	3.02
5	Qujin, Yunnan	2.81	43.47	6.29	20.93	1,522.30	3.13	3.02
6	Bozhou, Anhui	4.03	42.63	9.92	25.43	966.70	3.55	4.68
7	Bozhou, Anhui	4.47	43.98	10.38	27.54	646.20	4.10	4.95
8	Bozhou, Anhui	3.86	50.08	9.18	15.56	3,028.30	2.84	2.17
9	Bozhou, Anhui	4.32	48.90	6.53	19.08	1,383.30	3.52	3.48
10	Bozhou, Anhui	4.02	43.76	7.47	20.84	1,273.70	3.10	3.57
11	Bozhou, Anhui	4.16	50.28	7.38	21.52	744.50	3.75	4.10
12	Bozhou, Anhui	4.90	59.34	8.29	18.48	792.00	3.59	4.15
13	Bozhou, Anhui	3.59	43.59	6.02	23.94	986.30	2.93	2.72
14	Bozhou, Anhui	5.58	64.91	6.59	22.54	1,409.40	2.97	2.58
15	Bozhou, Anhui	4.89	51.37	7.22	21.85	1,035.80	3.31	3.17
16	Fuyang, Anhui	5.01	42.89	9.19	22.89	1,096.40	3.46	3.07
17	Fuyang, Anhui	3.65	45.41	15.75	21.29	881.40	3.88	3.72
18	Fuyang, Anhui	3.95	50.04	8.94	14.64	810.00	3.38	4.03
19	Fuyang, Anhui	4.48	42.53	9.43	22.78	890.30	2.96	4.37
20	Gaozhou, Guangdong	3.01	41.75	5.29	17.98	1,045.60	2.81	2.34
21	Gaozhou, Guangdong	3.07	42.56	5.92	14.86	780.40	2.56	2.39
22	Gaozhou, Guangdong	3.97	41.35	6.04	15.83	1,119.30	2.95	2.14
23	Deqin, Guangdong	3.09	40.82	11.88	21.77	601.30	4.00	4.05
24	Deqin, Guangdong	4.22	49.94	9.17	20.88	714.60	3.60	3.29
25	Deqin, Guangdong	4.79	44.69	8.07	22.37	1,047.70	2.77	2.18
26	Dazhou, Sichuan	4.44	41.28	9.70	24.69	985.20	3.04	4.59
27	Dazhou, Sichuan	2.76	44.84	6.37	24.46	1,181.60	3.17	4.10
28	Dazhou, Sichuan	3.19	56.85	10.01	13.29	853.40	3.61	3.70
29	Yibin, Sichuan	3.24	49.03	6.36	13.53	2,115.30	2.59	2.25
30	Yibin, Sichuan	2.30	50.36	8.05	18.44	1,473.90	3.07	2.80
31	Nanyang, Henan	3.66	48.57	7.42	19.02	1,161.70	2.10	3.65
Mean ± SD		3.80 ± 0.83	47.21 ± 5.81	8.26 ± 2.15	20.05 ± 3.76	1,151.46 ± 474.69	3.22 ± 0.44	3.40 ± 0.80
32	Bozhou, Anhui	11.20	65.89	5.64	21.08	736.30	1.91	7.62
33	Bozhou, Anhui	12.84	67.84	6.49	17.84	727.70	1.77	6.25
34	Bozhou, Anhui	11.86	61.96	5.79	21.98	663.90	1.94	6.87
35	Bozhou, Anhui	13.14	63.81	5.48	19.87	802.20	1.84	5.33
36	Bozhou, Anhui	12.02	64.75	6.59	20.14	715.80	1.96	5.90
37	Yulin, Guangxi	11.46	68.75	5.45	18.43	446.80	1.94	12.16
38	Anguo, Hebei	14.08	67.02	6.92	17.88	692.00	1.91	6.09
39	Nanyang, Henan	12.56	69.49	4.29	16.96	341.60	1.89	9.00
40	Yuzhou, Henan	20.73	71.05	5.35	13.05	404.70	1.98	9.53
Mean ± SD		13.32 ± 2.75^a^	66.73 ± 2.74^a^	5.78 ± 0.75^a^	18.58 ± 2.50	614.56 ± 169.89^a^	1.903 ± 0.06^a^	7.64 ± 2.21^a^

a *p* < 0.05 denotes a statistically significant difference compared with RPMPs.

Standards included mannose (Man), rhamnose (Rha), glucuronic acid (GlcA), galacturonic acid (GalA), glucose (Glc), galactose (Gal), and arabinose (Ara), all purchased from the National Institute for Food and Drug Control (China). 1-Phenyl-3-methyl-5-pyrazolone was purchased from Sigma (United States). Trifluoroacetic acid (TFA) was purchased from Oka (China). A Millipore Milli-Q Plus system was used to make deionized water (United States). All the other reagents and chemicals were of analytical grade.

### 2.2 Preparation of RPMPs and PPMPs

Each sample (5.0 g) was immersed in 100.0 mL of 80% ethanol solution for 1 h at 85°C. After filtration, the dry residues were extracted with water (1:20, m/v) in the bath for 2 h at 100°C. After centrifugation (5,000 rpm for 10 min), the supernatant was evaporated to 10.0 ml on a water bath, following which ethanol (95%, w/v) was added to the final concentration of 80% (v/v) for precipitation and kept overnight (>12 h) under 4°C. The precipitate was then collected by centrifugation and washed with ethanol. The precipitate was freeze-dried to obtain the crude polysaccharides (RPMPs and PPMPs). The yields were calculated according to the weight ratio of the freeze-dried polysaccharide to the dried powder.

### 2.3 Chemical Composition Determination

The polysaccharides from thirty-one raw PM and nine processed PM were obtained. The neutral sugar content was analyzed by the phenol-sulfuric acid method using Glc as the standard (DuBois et al., 1956). The uronic acid content was determined by a modified carbazole sulfate using GalA as the standard (McCready and McComb, 1953). The protein content was determined by Coomassie Brilliant Blue method using bovine serum albumin (BSA) as the standard (Bradford, 1976).

### 2.4 Determination of Molecular Weight and Polydispersity Index

The molecular weight (*M*w) and the polydispersity index (*PDI*) of polysaccharide samples were determined by high-performance size exclusion chromatography coupled with multi-angle laser light scattering and refractive index detector (HPSEC-MALLS-RID). The chromatographic signals were collected by Multi-angle Light Scattering Detectors (MALLS, DAWN HELEOS, Wyatt Technology Co., Santa Barbara, CA, United States) and RI detector (Shimadzu Company, Japan) in series. Each sample (10 mg) was dissolved in the mobile phase (1 ml) and then filtered through a 0.45 µm membrane. Two size exclusion columns Shodex SB-806 (300 mm × 7.8 mm, i. d.), and Shodex SB-804 (300 mm × 7.5 mm, i. d.) were used. The mobile phase included a 0.1 mol/L NaCl aqueous solution applied at a flow rate of 0.5 ml/min. An injection volume of 100 µL was used. Each sample was run for 50 min, and the temperature of the column was maintained at 40°C.

### 2.5 Monosaccharide Composition Analysis

The monosaccharide compositions of polysaccharide samples were determined by the precolumn derivation UPLC (PCD-UPLC) method. Each sample (1 mg/ml) was hydrolyzed with trifluoroacetic acid (TFA, 4 mol/L) at 120°C for 2 h. Then, TFA was removed by washing with methanol three times. Subsequently, the acid hydrolysates were derivatized with 1-Phenyl-3-methyl-5-pyrazolone (0.5 mol/L) at 70°C for 90 min. The injection volume was 2 μL and the samples were analyzed using a ZORBAX Eclipse XDB-C18 column (100 mm × 2.1 mm, 1.8 μm, Agilent, United States) with UV detection at 250 nm. The mobile phase consisting of acetonitrile and 0.125 mol/L KH_2_PO_4_ (v/v = 16:84, pH 6.9) was used at a flow rate of 0.3 ml/min.

### 2.6 *In vitro* Experiments

#### 2.6.1 Cell Culture

The Korean Cell Line Bank provided RAW 264.7 murine macrophage cells (Seoul, Republic of Korea). Cells were suspended in Dulbecco’s modified Eagle’s medium (DMEM; Gibco Inc, New York, United States) supplemented with 10% heat-inactivated fetal bovine serum (FBS) in an atmosphere of 5% CO_2_ at 37°C.

#### 2.6.2 Determination of Viability of RAW 264.7 Cells

The proliferation effects of RPMPs and PPMPs on RAW 264.7 cells were identified by using the MTT assay as previously described (Sun et al., 2019, 7). RAW 264.7 cells were seeded at 1 × 10^4^ cells/well in 96-well flat-bottom plates with medium, after which they (5,000 cells/well) were treated with various concentrations of RPMPs and PPMPs (25 μg/ml, 50 μg/ml, 100 μg/ml, 200 μg/ml, 400 μg/ml and 600 μg/ml) or lipopolysaccharides (LPS) (1 μg/ml) for 24 h. The absorbance (A) of each well was read at 490 nm using a microplate reader (Biochrom NanoVue Plus, United States), after which macrophage cell viability was calculated using the following equation: Macrophage cell viability (%) = [(A_t_-A_0_)/(A_t_-A_c_)] × 100, where A_t_ is the absorbance of the sample, A_c_ is the absorbance of the control group, and A_0_ is the absorbance of the blank group.

#### 2.6.3 Quantitative Real-Time Polymerase Chain Reaction Assay

The mRNA expression levels of inducible nitric oxide synthase (iNOS), interleukin 6 (IL-6), TNF-α (tumor necrosis factor-alpha), and total RNA were measured by Quantitative real-time polymerase chain reaction (qRT-PCR). Total RNA was isolated using TRIzol reagent, and RNA was transcribed to the cDNA using the RevertAid First Strand cDNA Synthesis Kit according to the manufacturer’s protocol. The qRT-PCR was performed using a multicolor detection system (Applied Biosystems, United States). The following sequences for PCR primers from 5′ to 3′ end were used: *IL-6*: forward, 5′-CCA​TGC​CAT​GGA​AGA​TTC​CA AAGATGTAG-3′, *IL-6*: reverse, 5′-CTC​GCT​CGA​GCT​ACA​TTT​GCC​GAA​GAG​CC C-3’; TNF-α: forward, 5′-CAT​GAT​CCG​GGA​CGT​GGA​G-3′, TNF-α: reverse, 5′-CG ATC​ACT​CCA​AAG​TGC​AGC-3’; *iNOS*: forward, 5′-CAC​CTA​CCC​ACC​CCT​ACA​AA -3′, *iNOS*: reverse, 5′-CAG​CCA​ACG​TGG​AGA​CTA​CC-3’; Glyceraldehyde-3- phosphate dehydrogenase (*GAPDH*) was used as the internal reference gene. The expression levels concerning the control were estimated by calculating ΔΔCt and subsequently analyzed using the 2^−ΔΔCt^ method.

### 2.7 Statistical Analysis

The results were expressed as mean ± standard deviation (SD). Statistical differences between groups were assessed by Student’s t-test. The PCD-UPLC fingerprints were generated by ChemPattern software (Chemmind Technologies Co, Ltd, Beijing) and analyzed by similarity analysis (SA), principal component analysis (PCA), and partial least squares regression discriminant analysis (PLS-DA).

## 3 Results and Discussion

### 3.1 The Components and Yields of RPMPs and PPMPs

The crude water-soluble polysaccharides were extracted from raw PM (1–31) and processed PM (32–40). The yields, neutral sugar, uronic acid, and protein contents of 40 polysaccharide samples are shown in Table 1. The range of RPMPs yields was 2.05–5.58%, and the range of PPMPs yields was 11.20–20.73%. The contents of neutral sugar, uronic acid, and protein of RPMPs were 40.39–64.91%, 5.29–15.75%, and 13.20–25.43%, respectively. The contents of neutral sugar, uronic acid, and protein of PPMPs were 61.96–71.05%, 4.29–6.59%, and 13.05–21.98%, respectively. The results showed there were significant differences between RPMPs and PPMPs in terms of yields, neutral sugar content, and uronic acid content (*p* < 0.05), which could be due to various reasons. It could be that the high temperature steaming during the processing enhances the permeability of the cells and promotes the leaching of polysaccharides. Moreover, the polysaccharides in the black bean juice might remain on the PM during the processing, as they are extracted together with the PPMPs. In addition, it was also found that the PM steaming process was involved in the Maillard reaction, which may explain the significant difference in the yields of RPMPs and PPMPs (Liu et al., 2009).

### 3.2 HPSEC-MALLS-RID Analysis

**FIGURE 1:**
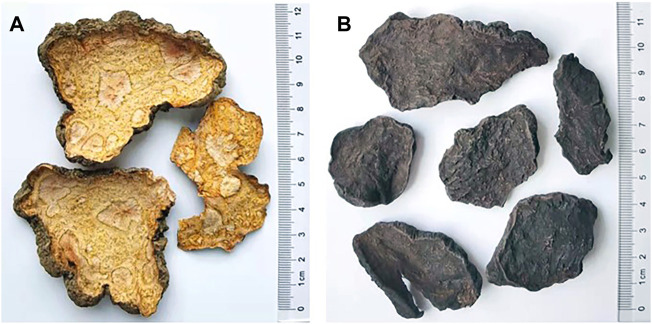
The roots of *Polygonum multiflorum* Thunb **(A)** raw product, and **(B)** processed product.

Polysaccharides are a kind of macromolecular polymers, and the molecular chain of polysaccharides could be reflected by *M*w and *PDI*. It has also been found that the structure and physiological activities of polysaccharides are closely related to their *M*w (Hou et al., 2012). HPSEC-MALLS-RID is an effective and powerful method for analyzing the *M*w and *PDI* of polysaccharides from TCM. It is also considered to be beneficial for the discrimination and quality control of polysaccharides from TCM. Therefore, to further analyze the differences between RPMPs and PPMPs, the HPSEC chromatograms and the *M*w of 40 polysaccharide samples were compared. Table 1 summarized the *M*w and *PDI* of RPMPs and PPMPs from different regions and markets. Generally, the *PDI* is a measure of the distribution of *M*w in a given polymer sample, which is often denoted as *PDI* = *M*w/*M*n. Results showed that the *M*w of RPMPs and PPMPs were not similar, ranging from 646.20 to 3,028.30 kDa (RPMPs) and 341.60 to 802.20 kDa (PPMPs), respectively. The average *M*w of thirty-one RPMPs was 1,151.46 kDa, while the average *M*w of nine PPMPs was 606.98 kDa. The difference was also found in the *PDI* of RPMPs and PPMPs, while the *PDI* of thirty-one batches of RPMPs ranged from 2.10 to 4.10, as well as the *PDI* of nine batches ranged from 1.77 to 1.98, thus suggesting that the *M*w distribution of PPMPs was narrower and more concentrated than that of RPMPs (Figure 1).

**FIGURE 2:**
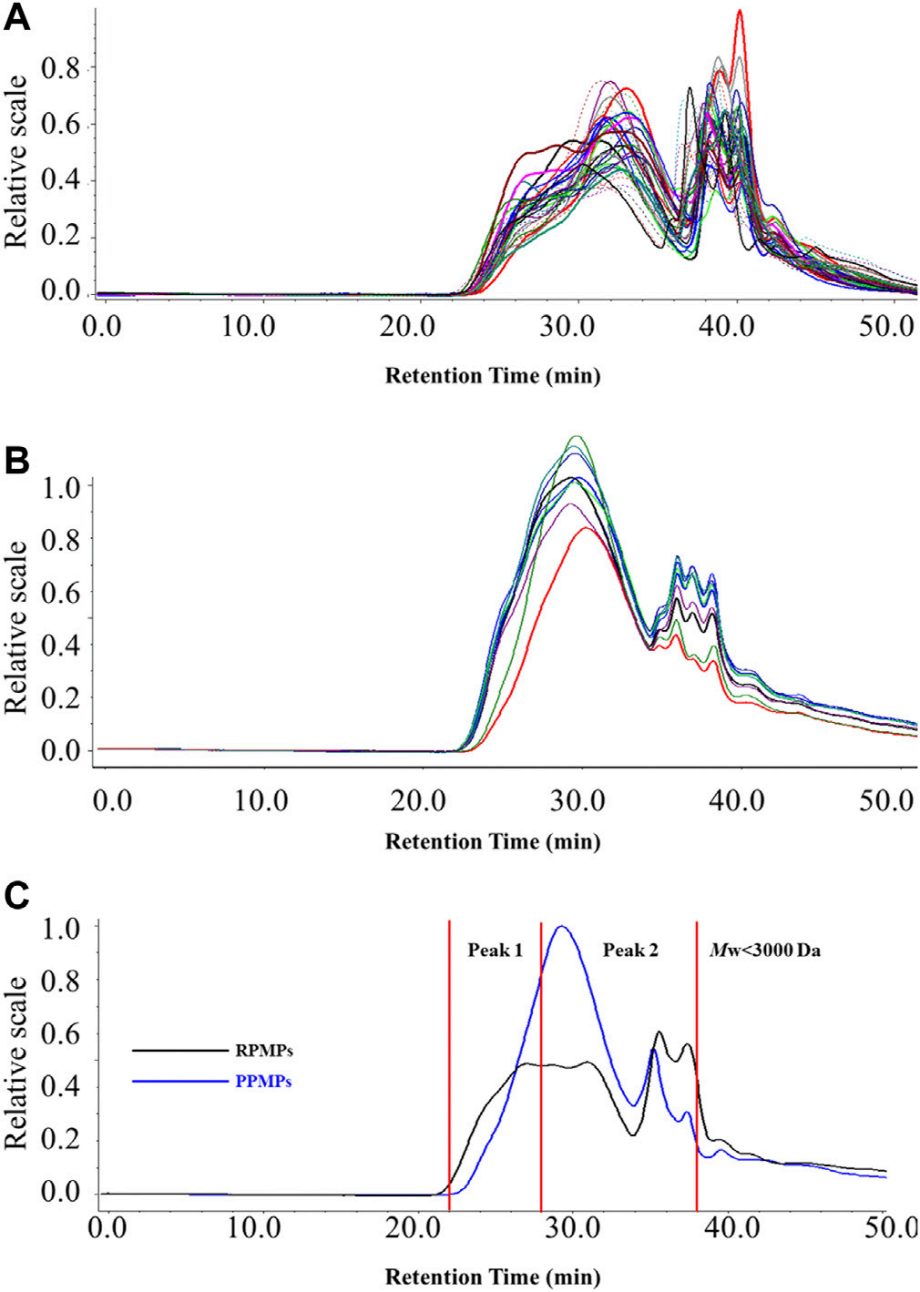
The HPSEC-MALLS-RID chromatograms of 40 polysaccharide samples **(A)** RPMPs **(B)** PPMPs, and **(C)** the representative chromatograms of RPMPs and PPMPs.

Subsequently, the retention time corresponding to the dextran standard with *M*w 820 kDa (t_R_ = 28.01 min) was used to divide the main peak into two parts (peak 1, peak 2). As shown in Figure 2C and Table 1, peak 2 of RPMPs accounted for a lower proportion of the main peak than peak 1. The results revealed that the low *M*w part (peak 2) of PPMPs was more than that of RPMPs, that is, the content of polysaccharides with low *M*w increased after processing. It is speculated that the polysaccharides from raw PM might be degraded into polysaccharides with lower *M*w due to the extended period of high temperature during the processing, which was similar to the results reported by Sun et al. (2020). They compared the physicochemical properties of water-soluble polysaccharides from raw and wine-processed *Polygonatum sibiricum*, and concluded that most macromolecule polysaccharides were degraded to small molecular polysaccharides during the processing.

### 3.3 Monosaccharide Composition Analysis of RPMPs and PPMPs

**FIGURE 3:**
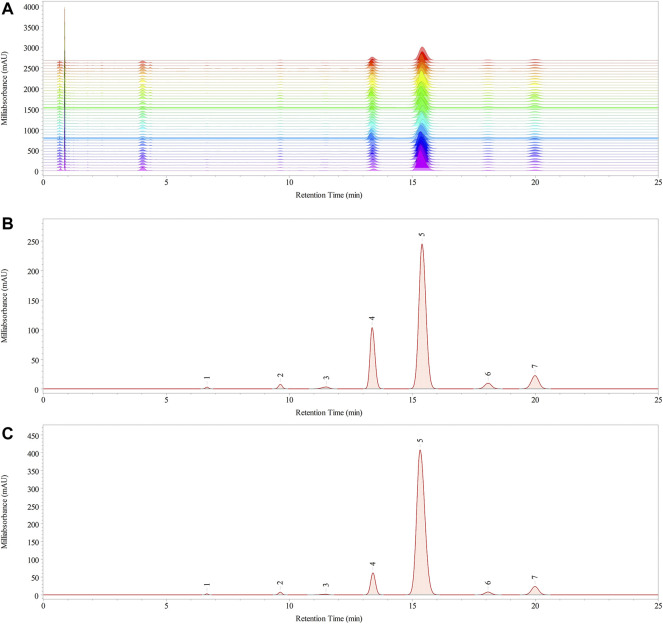
The chromatograms of 40 polysaccharide samples **(A)** PCD-UPLC fingerprints, and the PCD-UPLC standard referential fingerprint of RPMPs **(B)** and PPMPs **(C)**.

The monosaccharide composition of polysaccharides is an essential parameter for evaluating the structural features of the samples. The monosaccharide compositions of RPMPs and PPMPs were analyzed based on PCD-UPLC following acid hydrolysis. The PCD-UPLC chromatograms from 40 polysaccharide samples are shown in Figure 3A. The results showed that RPMPs and PPMPs samples from different regions and markets all consisted of seven types of monosaccharides. The first to the seventh peaks represented Man, Rha, GlcA, GalA, Glc, Gal, and Ara, respectively, and Glc was the main monosaccharide in RPMPs and PPMPs samples.

Although the monosaccharide composition of RPMPs and PPMPs was the same, the molar ratios of monosaccharides between RPMPs and PPMPs were significantly different, especially in the molar ratio of Glc (*p* < 0.05). We found that the molar ratio of Glc/GalA of PPMPs (16.42 ± 3.94) was significantly higher than that of RPMPs (6.50 ± 2.08), as shown in Table 2. The results showed that the molar ratio of Glc/GalA could be used as an important marker to distinguish RPMPs and PPMPs.

**TABLE 2:** The monosaccharide composition of 40 polysaccharide samples.

Codes	Man	Rha	GlcA	GalA	Glc	Gal	Ara
1	0.02	0.06	0.04	1.00	8.34	0.16	0.34
2	0.01	0.07	0.03	1.00	7.57	0.16	0.38
3	0.01	0.08	0.03	1.00	3.72	0.14	0.35
4	0.02	0.06	0.04	1.00	7.59	0.19	0.34
5	0.02	0.07	0.06	1.00	9.74	0.18	0.32
6	0.02	0.06	0.04	1.00	4.26	0.14	0.29
7	0.01	0.06	0.04	1.00	4.52	0.14	0.34
8	0.01	0.04	0.02	1.00	4.65	0.07	0.18
9	0.01	0.07	0.04	1.00	7.26	0.11	0.34
10	0.01	0.06	0.04	1.00	5.87	0.12	0.29
11	0.01	0.06	0.03	1.00	7.00	0.14	0.31
12	0.01	0.06	0.04	1.00	9.14	0.15	0.33
13	0.01	0.06	0.03	1.00	8.08	0.13	0.32
14	0.01	0.08	0.03	1.00	6.67	0.14	0.38
15	0.01	0.07	0.03	1.00	5.85	0.14	0.34
16	0.01	0.05	0.04	1.00	5.84	0.11	0.27
17	0.01	0.06	0.03	1.00	2.80	0.12	0.34
18	0.01	0.05	0.05	1.00	6.10	0.13	0.26
19	0.03	0.08	0.04	1.00	3.70	0.20	0.40
20	0.01	0.06	0.10	1.00	8.83	0.12	0.30
21	0.01	0.06	0.11	1.00	8.95	0.14	0.36
22	0.01	0.06	0.08	1.00	6.25	0.12	0.39
23	0.01	0.09	0.04	1.00	2.62	0.21	0.41
24	0.01	0.08	0.03	1.00	4.75	0.17	0.45
25	0.01	0.08	0.06	1.00	4.80	0.15	0.48
26	0.07	0.08	0.07	1.00	7.57	0.52	0.35
27	0.02	0.06	0.06	1.00	6.97	0.66	0.30
28	0.01	0.07	0.03	1.00	6.23	0.16	0.34
29	0.01	0.11	0.06	1.00	11.69	0.34	0.50
30	0.01	0.07	0.04	1.00	5.80	0.13	0.32
31	0.01	0.09	0.05	1.00	8.23	0.21	0.50
Mean ± SD	0.01 ± 0.01	0.07 ± 0.01	0.05 ± 0.02	—	6.50 ± 2.08	0.18 ± 0.12	0.35 ± 0.07
32	0.02	0.12	0.09	1.00	21.87	0.21	0.56
33	0.01	0.11	0.06	1.00	17.40	0.25	0.72
34	0.02	0.12	0.05	1.00	13.73	0.25	0.77
35	0.02	0.12	0.05	1.00	12.17	0.23	0.73
36	0.02	0.13	0.04	1.00	13.72	0.24	0.81
37	0.04	0.14	0.06	1.00	16.23	0.33	0.50
38	0.02	0.12	0.06	1.00	14.46	0.24	0.74
39	0.03	0.12	0.10	1.00	23.73	0.21	0.53
40	0.02	0.12	0.06	1.00	14.46	0.24	0.74
Mean ± SD	0..02 ± 0.01	0.12 ± 0.01^a^	0.06 ± 0.01	—	16.42 ± 3.94^a^	0.24 ± 0.04	0.68 ± 0.11^a^

a *p* < 0.05 denotes a statistically significant difference compared with RPMPs.

### 3.4 PCD-UPLC Fingerprints and Chemometric Analysis

#### 3.4.1 SA of the PCD-UPLC Fingerprints

The PCD-UPLC fingerprints of the 40 polysaccharide samples are displayed in Figure 3A. The correlation coefficients method and cosine (cos θ) method were used to evaluate the similarity of the monosaccharide composition of 40 polysaccharide samples. The similarity values were calculated based on the common pattern, which was generated by Chempattern software based on the PCD-UPLC chromatograms of PPMPs. The value of cos θ ranged from 0.9762 to 0.9996, and the value of correlation coefficients ranged from 0.9737 to 0.9996. Results showed that the similarity value of all 40 samples was >0.95, which indicated that all samples were highly similar, and there were no obvious differences among RPMPs and PPMPs from different locations (*p* > 0.05). In addition, the standard fingerprint of RPMPs and PPMPs were also established, as shown in Figures 3B,C.

#### 3.4.2 PCA of the PCD-UPLC Fingerprints

**FIGURE 4:**
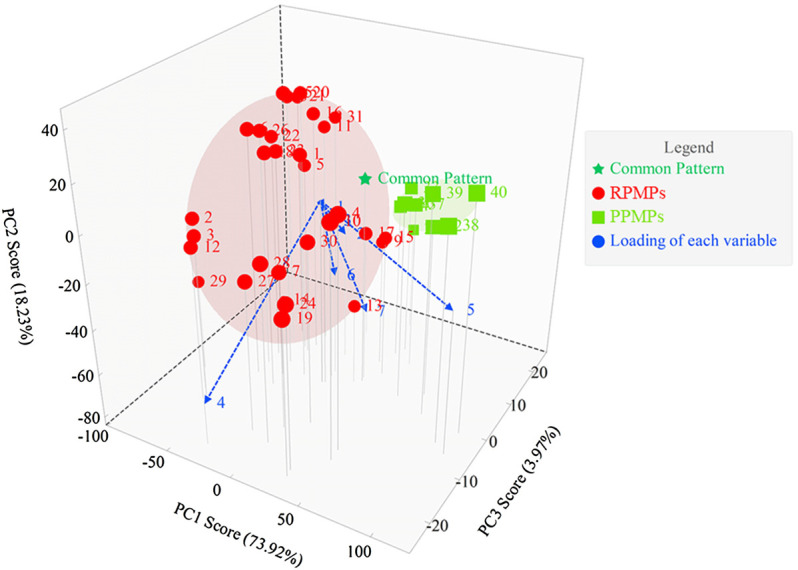
The score plot and loading plot of PCA from PCD-UPLC fingerprints.

PCA is the most common method for data analysis in multivariate statistical analysis. PCD-UPLC fingerprints were analyzed by PCA to find out the index of distinguishing RPMPs and PPMPs. The data matrix of relative peak areas of the seven characteristic monosaccharide peaks was worked by ChemPattern software. The score plot and the loading of PCA are shown in Figure 4. Results showed that the first three PCs explained 73.92, 18.23, and 3.97% of the variance, respectively, accounting for 96.12% of the total variance, which could reflect the vast majority of the original chromatographic information of the samples. Three main factors could be calculated by the equations as follows:

PC1=0.02∗X−0.02∗X2+0.02∗X3−0.28∗X4+0.96∗X5+0.02∗X6−0.02∗X7

PC2=0.03∗X1−0.23∗X2−0.10∗X3−0.59∗X4−0.18∗X5−0.34∗X6−0.66∗X7

PC3=0.01∗X1+0.29∗X2−0.08∗X3−0.75∗X4−0.21∗X5+0.12∗X6+0.55∗X7

In the equations, X1 to X7 were Man, Rha, GlcA, GalA, Glc, Gal, and Ara, respectively. PCA could classify RPMPs and PPMPs into two categories. As shown in Figure 4, the loading of each variable shows that peak four and peak five had a great influence on the identification of RPMPs and PPMPs, which indicates that Glc and GalA could be chosen as the markers to distinguish raw and processed PM.

#### 3.4.3 PLS-DA of the PCD-UPLC Fingerprints

**FIGURE 5:**
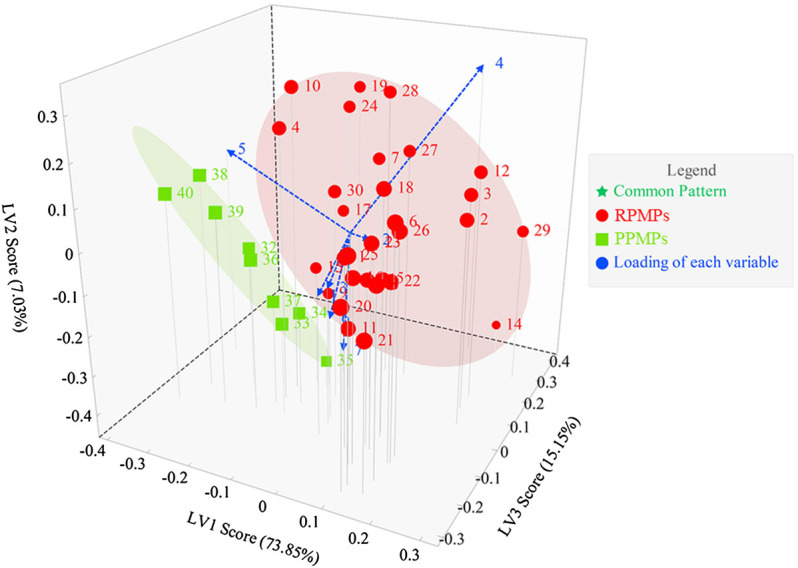
The score plot and loading plot of PLS-DA from PCD-UPLC fingerprints.

PLS-DA is an analytical method with supervised pattern recognition, which combines the advantages of multivariate linear regression and PCA. The 40 chromatograms were pretreated, and PLS-DA were analyzed by their pattern. As shown in Figure 5, results showed that PLS-DA could effectively separate RPMPs and PPMPs. As with PCA, peak 4 and peak 5 contribute significantly to the differentiation of RPMPs and PPMPs. The contents of these two monosaccharides in analyzed samples were higher, which verified the results of PCA and PLS-DA. This also suggested that the Glc and GalA in polysaccharides from PM might have changed after processing.

### 3.5 Immunomodulatory Activities of RPMPs and PPMPs

The RPMPs and PPMPs (sample 31 and sample 39) used in this experiment were derived from the same batch of PM. Also, its physicochemical properties were in accordance with the characteristics of common polysaccharides from raw and processed PM.

#### 3.5.1 Effect of RPMPs and PPMPs on Cell Viability of RAW 264.7 Cells

**FIGURE 6:**
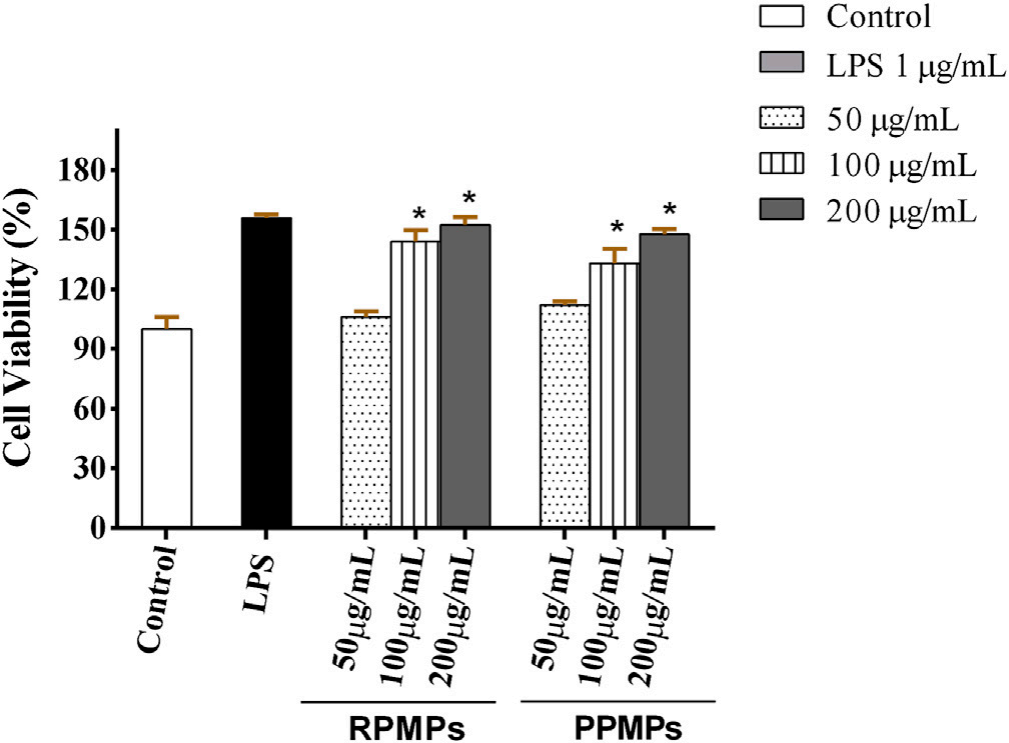
Effects of RPMPs and PPMPs on the viability of RAW 264.7 cells. Results are represented as mean ± SD, *n* = 6. *p < 0.05 denotes a statistically significant difference between the treated and control groups.

Macrophages are vital immune cells that serve multiple functions. To resist invading pathogens, macrophages can phagocytize and kill harmful bacteria, as well as produce and emit chemokines and cytokines (Mingzhu Zhang et al., 2018; Yang et al., 2020). RAW 264.7 cells are commonly used cell models to evaluate the immune activity of polysaccharides *in vitro*. The viability of RAW 264.7 cells treated by RPMPs and PPMPs was investigated through the MTT assay. RPMPs and PPMPs with a concentration of 25–600 μg/ml were not toxic to RAW 264.7 macrophages. Therefore, the concentration of RPMPs and PPMPs used in this study was lower than 600 μg/mL. As shown in Figure 6, treatment with different doses of RPMPs and PPMPs significantly enhanced RAW 264.7 cells (*p* < 0.05). The proliferation of RAW 264.7 cells was similarly aided by this therapy in a dose-dependent manner. When the concentration of RPMPs and PPMPs was 200 μg/ml, the cell viability reached the maximum value of 152.6 and 147.7%, respectively, which was close to that of the LPS treatment group (156.1%). In addition, there was no significant difference between RPMPs and PPMPs at the level of 200 μg/ml (*p* > 0.05).

#### 3.5.2 Effect of RPMPs and PPMPs on Cell Viability of RAW 264.7 Cells

Activated macrophages do induce not only the expression but also enhance the level of cytokines (such as TNF-α and IL-6) (Zhan et al., 2020). NO, TNF-α and IL-6 are important bioactive molecules in the human body. NO can activate macrophages and kill pathogenic microorganisms and tumor cells (Ren et al., 2017). IL-6 plays a role in the pathophysiology of inflammatory and immunological disorders as a key regulator of the host’s defensive response (Wang et al., 2015). TNF-α may activate macrophages, increase different functional responses, and induce the expression of antitumor and immunomodulatory mediators (He et al., 2012).

**FIGURE 7:**
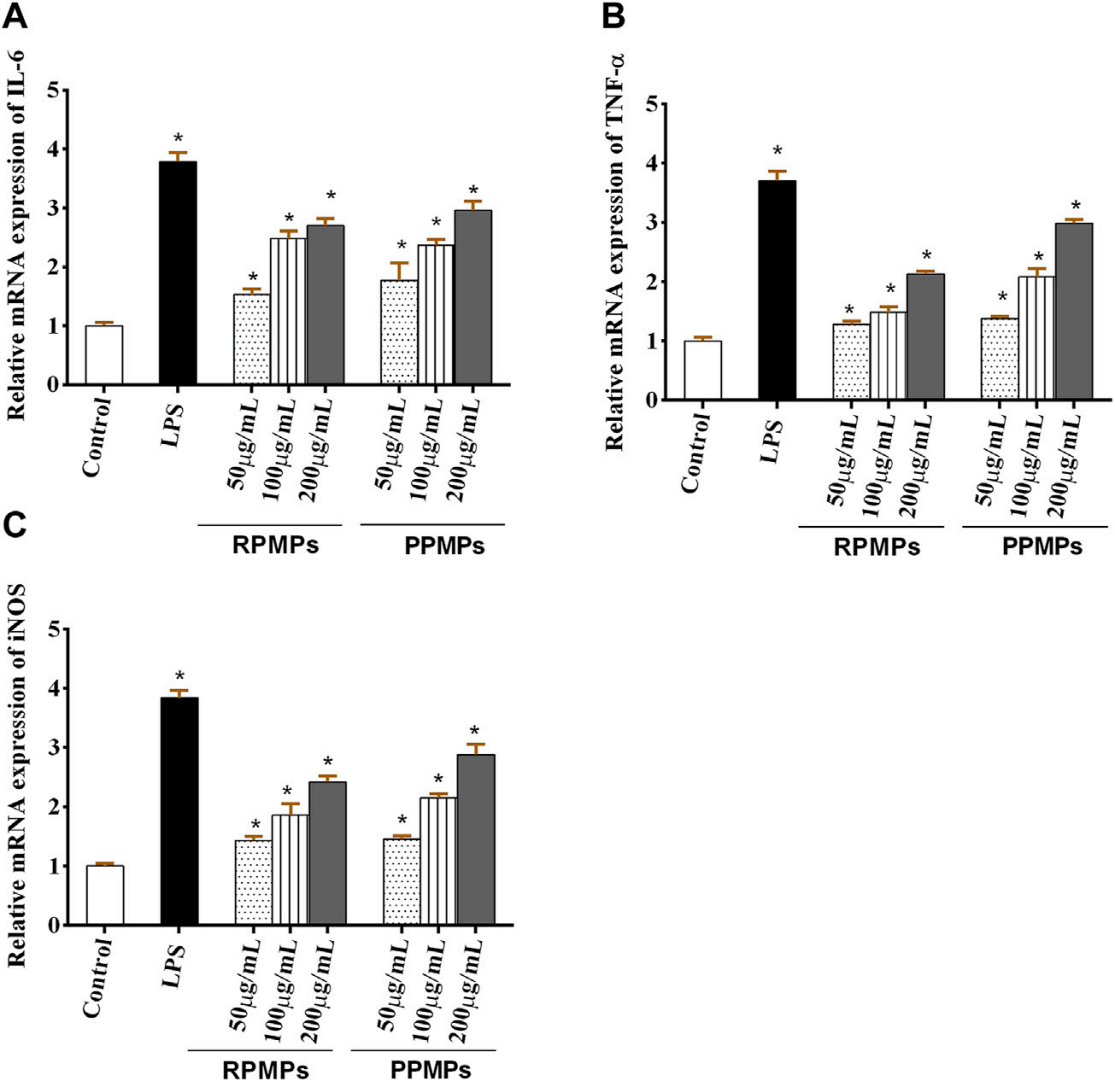
Effects of RPMPs and PPMPs on the mRNA expression of IL-6 **(A)**, TNF-α **(B)**, and iNOS **(C)** in RAW 264.7 cells. *p < 0.05 versus the control. The data are presented as mean ± SD (*n* = 3).

Meanwhile, the level of TNF-α, which is related to the production of NO, can induce the up-regulation of iNOS, resulting in the release of NO. Several previous studies investigated the immune modulation effect of polysaccharides by using RAW264.7 macrophages as a cell model *in vitro*. To further evaluate the immunostimulatory effect of RPMPs and PPMPs, the effects of RPMPs and PPMPs on NO, IL-6 and TNF-α at the molecular level, the expression of iNOS, IL-6, and TNF-α were determined by qRT-PCR. As shown in Figure 7, RPMPs and PPMPs promoted the mRNA expression of IL-6, TNF-α, and iNOS in a dose-dependent manner compared with the control group. The high-dose experimental group (200 μg/ml) of RPMPs and PPMPs increased 2∼3 fold the mRNA expression levels of iNOS, IL-6, and TNF-α (*p* < 0.05) compared with the control group. These results suggested that RPMPs and PPMPs could promote the secretion of NO, IL-6, and TNF-α in RAW 264.7 cells, where the effect of the PPMPs was greater than that of the RPMPs (*p* < 0.05).

### 3.6 The Change of Physicochemical Properties and Immune Activity of polysaccharides After Processing

PM is a well-known traditional Chinese medicine with a long medicinal history. Polysaccharides are an important component of raw and processed PM. In this study, there were significant differences between RPMPs and PPMPs in yields, neutral sugar contents, uronic acid contents, *M*w, and the molar ratio of Glc/GalA (*p* < 0.05). These results indicated that polysaccharides in PM changed after processing. This change may be due to the permeability of the cell wall increased when water or black bean juice was immersed into PM at high temperature, and black bean juice also flowed into PM during processing. In addition, the high temperature during processing may lead to the degradation of the most macromolecular polysaccharides into small ones, which has also been previously reported (Sun et al., 2020). The darker color and lower *M*w of PPMPs might be related to the Maillard reaction during the processing.

It was proposed that the monosaccharide composition, uronic acid content, configuration, as well as *M*w, were crucial for the immune activity. Recent studies have shown that polysaccharides with different *M*w have different biological activities (Xu et al., 2015). For example, Li et al. (2020) obtained three polysaccharide fractions APS-Ⅰ (>2000 kDa), APS-Ⅱ (about 10 kDa), and APS-Ⅲ (about 300 Da), and evaluated their immune activity, revealing that APS-Ⅱ (about 10 kDa) with moderate *M*w had better immune activity. Hou et al. (2012) pointed out that with the decrease in *M*w of polysaccharides, their water solubility increased and viscosity decreased, which promoted the movement of polysaccharides *in vivo* and increased the biological activity. Qi and Kim (2018) suggested that the *M*w had an important impact on the immunomodulatory effects of polysaccharides from the green alga *Chlorella ellipsoidea*. All the above studies showed that polysaccharide with low *M*w was an important component contributing to immunomodulatory. In our study, both RPMPs and PPMPs had regulatory effects on RAW 264.7 cells, and PPMPs with lower *M*w had significantly better immune activity than RPMPs. It is presumed that the immunomodulatory activity of polysaccharides from PM is related to *M*w. However, homogeneous polysaccharides with different molecular weights need to be prepared to further verify the above inference.

## 4 Conclusion

In this study, the physicochemical properties and immunomodulatory activities of polysaccharides from raw and processed PM were determined and compared. Results showed that there were significant differences in the yields, *M*w, and the molar ratio of Glc/GalA between RPMPs and PPMPs, revealing that the polysaccharides from PM changed after processing. Also, these indexes could be used to distinguish raw and processed PM. In addition, both RPMPs and PPMPs had immunomodulatory activity, and the activity of PPMPs was superior to that of RPMPs, which was consistent with the ancient processing theory that processed PM has a tonic effect. This study provides a reference for improving the quality control standard of PM and the study of its immunomodulatory activity.

## Data Availability

The original contributions presented in the study are included in the article/Supplementary Material, further inquiries can be directed to the corresponding author.
